# Evaluating the fit and performance of flat-fold, cup, and three-panel respirators among Thai healthcare personnel

**DOI:** 10.3389/fpubh.2025.1561571

**Published:** 2025-04-28

**Authors:** Kampanat Wangsan, Ratana Sapbamrer, Wachiranun Sirikul, Wuttipat Kiratipaisarl, Krongporn Ongprasert, Pheerasak Assavanopakun, Vithawat Surawattanasakul, Amornphat Kitro, Jinjuta Panumasvivat, Amnart Wongcharoen

**Affiliations:** ^1^Department of Community Medicine, Faculty of Medicine, Chiang Mai University, Chiang Mai, Thailand; ^2^Environmental and Occupational Medicine Excellence Center (EnOMEC), Faculty of Medicine, Chiang Mai University, Chiang Mai, Thailand; ^3^Health Promotion Unit, Maharaj Nakorn Chiang Mai Hospital, Faculty of Medicine, Chiang Mai University, Chiang Mai, Thailand

**Keywords:** quantitative fit test, respiratory protection, filtering facepiece, respirator, N95, facial anthropometric, health personnel

## Abstract

**Introduction:**

Healthcare personnel (HCP) face high risks of airborne infections, including coronavirus disease 2019 (COVID-19), tuberculosis, and measles. Filtering facepiece respirators (FFRs) are critical for protection but require an adequate fit for effectiveness. Limited studies have explored the fit performance of different FFR designs in Southeast Asian populations. This study evaluates fit factors and pass rates of flat-fold, cup-shaped, and three-panel flat-fold respirators among Thai HCP and examines the influence of facial anthropometry on fit outcomes.

**Methods:**

A cross-sectional study was conducted with 223 HCP at a university hospital in Chiang Mai, Thailand. Quantitative fit testing of three NIOSH-certified N95 respirators—flat-fold, cup-shaped, and three-panel flat-fold—was performed using a TSI Portacount Pro+ 8,038 device. The Occupational Safety and Health Administration (OSHA) Condensation Nuclei Counter protocol, comprising bending, talking, and head movement exercises, was followed. Fit factors, calculated as the harmonic mean, required a passing threshold of ≥100. Twenty-two facial anthropometric dimensions were also measured. Statistical analyses included the Kruskal–Wallis test, Fisher’s exact test, and logistic regression.

**Results:**

Pass rates were 5.4% for flat-fold respirators (median fit factor [FF]: 25), 51.1% for cup-shaped models (median FF: 104), and 82.5% for three-panel flat-fold designs (median FF: 191), with significant differences (*p* < 0.001). The three-panel flat-fold maintained FF values near 200 across exercises. Anthropometric predictors varied by FFR type: head length (Adj. OR: 1.16) and nose length (Adj. OR: 1.28) influenced flat-fold models, while nasal bridge breadth (Adj. OR: 1.11) affected cup-shaped models.

**Conclusion:**

The three-panel flat-fold respirator exhibited superior adaptability, highlighting its potential as the preferred choice for Thai HCP. The low pass rate of flat-fold designs underscores the need for region-specific respirator designs. Findings emphasize the importance of localized fit testing and the development of regional fit test panels to enhance protection. Further research is needed to explore fit retention, comfort, and usability in real-world conditions.

## 1 Introduction

Healthcare personnel (HCP) are at a heightened risk of exposure to respiratory infections such as tuberculosis, measles, and COVID-19, which are primarily transmitted via droplet and airborne routes (1). To mitigate these risks, the use of well-fitting respiratory protection equipment is critical. The COVID-19 pandemic has underscored the significance of respiratory protection, with numerous studies demonstrating its efficacy in reducing respiratory infection rates in both public and healthcare settings (2, 3). However, the effectiveness of respirators depends not only on their filtration efficiency but also on their fit (4). Poor fit compromises the protective factor, allowing for inward leakage and diminishing overall effectiveness. Thus, respirator selection and fit-testing are pivotal in ensuring maximum protection (5, 6). The National Institute for Occupational Safety and Health (NIOSH) emphasizes the necessity of fit testing to confirm that respirators provide adequate protection to individual users. Fit-testing evaluates whether a particular respirator model is suitable for an individual’s facial structure, taking into account compatibility and minimizing leakage (5). As respirators vary widely in type, shape, and design, healthcare settings commonly employ various filtering facepiece respirators (FFRs) such as cup-shaped, duckbill, flat-fold, and multi-panel flat-fold designs (7). Each shape may offer a distinct user experience and fit factor, which can influence overall protection. Fit tests are essential for selecting a respirator that properly fits the wearer (8). It is crucial to conduct a fit test on FFRs before their use. Despite this, many workers in Thailand still rely on respirators that have not been chosen based on a fit test.

Facial anthropometry, sex, age, ethnicity, weight, and experience of the wearer are factors known to influence the fit factor (9–15). NIOSH recommends bivariate anthropometric dimensions, including face length and face width, for fit assessments (11). However, other studies suggest that dimensions such as face length and lip length may be more relevant for half-face respirators (12). Although some previous studies have examined fit performance and facial anthropometrics among Asian regions, such as China, the Republic of Korea, Malaysia, and Iran (13–19), there remains a significant knowledge gap among the Thai population. Given the diversity of facial anthropometry across different Asian populations, findings from other regions may not be directly applicable to Thailand. Understanding the specific fit characteristics of Thai HCP is essential for ensuring optimal respiratory protection.

To address this gap, this study evaluates the fit factors of various FFR designs and their pass rates among healthcare personnel in a Thai university hospital setting. Additionally, it provides preliminary data on Thai anthropometric dimensions, which remain under-researched. These findings may help inform FFR selection criteria and support the design of respirators tailored to the Thai population, addressing the current challenges in respirator compatibility. We hope the results will offer optimal protection, ensuring the safety of frontline workers against respiratory threats.

## 2 Method

### 2.1 Participants and data collection

Participants were recruited through voluntary response sampling from the University Hospital’s HCP. The inclusion criteria were HCP at risk of respiratory infection. The exclusion criteria included participants who intended to quit the study, had health conditions restricting the use of tight-fitting respirators, or had facial hair. Since facial hair can interfere with the respirator seal, full beards or styles crossing the sealing surface were excluded, in accordance with OSHA 29CFR 1910.134(g) (1)(i)(A) and NIOSH guidance (20, 21). Only participants with facial hairstyles deemed acceptable per the CDC/NIOSH (e.g., trimmed mustaches not interfering with the seal) were included. The study was conducted in accordance with the principles outlined in the Declaration of Helsinki and received approval from the Research Ethics Committee, Faculty of Medicine, Chiang Mai University, Thailand (Study Code: COM-2564-08382). All participants provided written informed consent before their inclusion in the study. The sample size was pre-determined for a fixed-effect, omnibus one-way ANOVA or Kruskal–Wallis test to assess differences in fit factor scores across the three mask types. G*Power software (version 3.1.9.7) was used, specifying a standardized effect size of 0.25, an alpha level of 0.05, and a power of 0.9. The calculated sample size is at least 207 samples. A total of 223 participants participated in the study, with no volunteers excluded. Basic characteristic data of participants, including age, sex, working information, health condition, history of facial surgery, and denture, were collected via questionnaires. Each participant was tested with three FFRs during one visit to the laboratory. Prior to testing, all participants received training from qualified staff on the correct use of respirators.

### 2.2 Respirators

A total of three NIOSH-certified N95s available in Thailand and commonly used in the medical industry, especially by HCP, were selected for this study. The selected FFRs represented three distinct designs: flat-fold, cup-shaped, and three-panel flat-fold. The three-panel flat-fold FFR used in this study was labeled as one-size-fits-all. The flat-fold and cup-shaped FFRs did not indicate sizing options, and only one standard size per model was available, consistent with routine hospital supply. Each participant underwent a fit test with all three FFR models.

### 2.3 Fit test protocol

The quantitative fit testing process was carried out using quantitative respirator fit testers (TSI Portacount Pro+ 8,038, USA), following a modified Condensation Nuclei Counter (CNC) protocol as outlined in OSHA 29CFR 1910.134 (20). This protocol comprised four exercises—bending, talking, turning the head side-to-side, and moving the head up and down.

The overall fit factor was calculated using the harmonic mean of the fit factors from each exercise. A test was considered successful if the overall fit factor met or exceeded the minimum threshold of ≥100.

Fit factor calculations were based on the following:

Calculation of fit factor (22).


$$FF=\frac{CB+CA}{2\;CR}$$

CB: Particle concentration in the ambient sample before the respirator sample.

CA: Particle concentration in the ambient sample after the respirator sample.

CR: Particle concentration inside the respirator sample.

The overall fit factor was derived as follows:


$$\mathrm{Overall}\;\mathrm{Fit}\;\mathrm{Factor}=\frac{1}{\frac{1}{FF1}+\frac{1}{FF2}+\frac{1}{FF3}+\frac{1}{FF4}}$$

Where:

FFx = Fit factor for a test cycle.

The fit tester was prepared before each test by thoroughly cleaning the testing tube and performing a daily system check using the Portacount device. To ensure sufficient ambient particles for accurate measurements, a controlled particle generation method was employed when necessary. This involved introducing a salt aerosol or ambient particle enhancer, following OSHA and NIOSH fit-testing guidelines, to maintain an adequate particle concentration within the test environment (20).

The test was conducted in a closed room (15 m^2^) with an air temperature of 25°C and humidity of approximately 60–70%.

To ensure accurate results, participants were required to be in good physical health and to refrain from smoking, eating, or drinking for at least 30 min before the test. Additionally, all participants received training on proper respirator donning and were assessed by well-trained occupational health staff before testing (23, 24).

### 2.4 Anthropometric measurement

**Figure 1:**
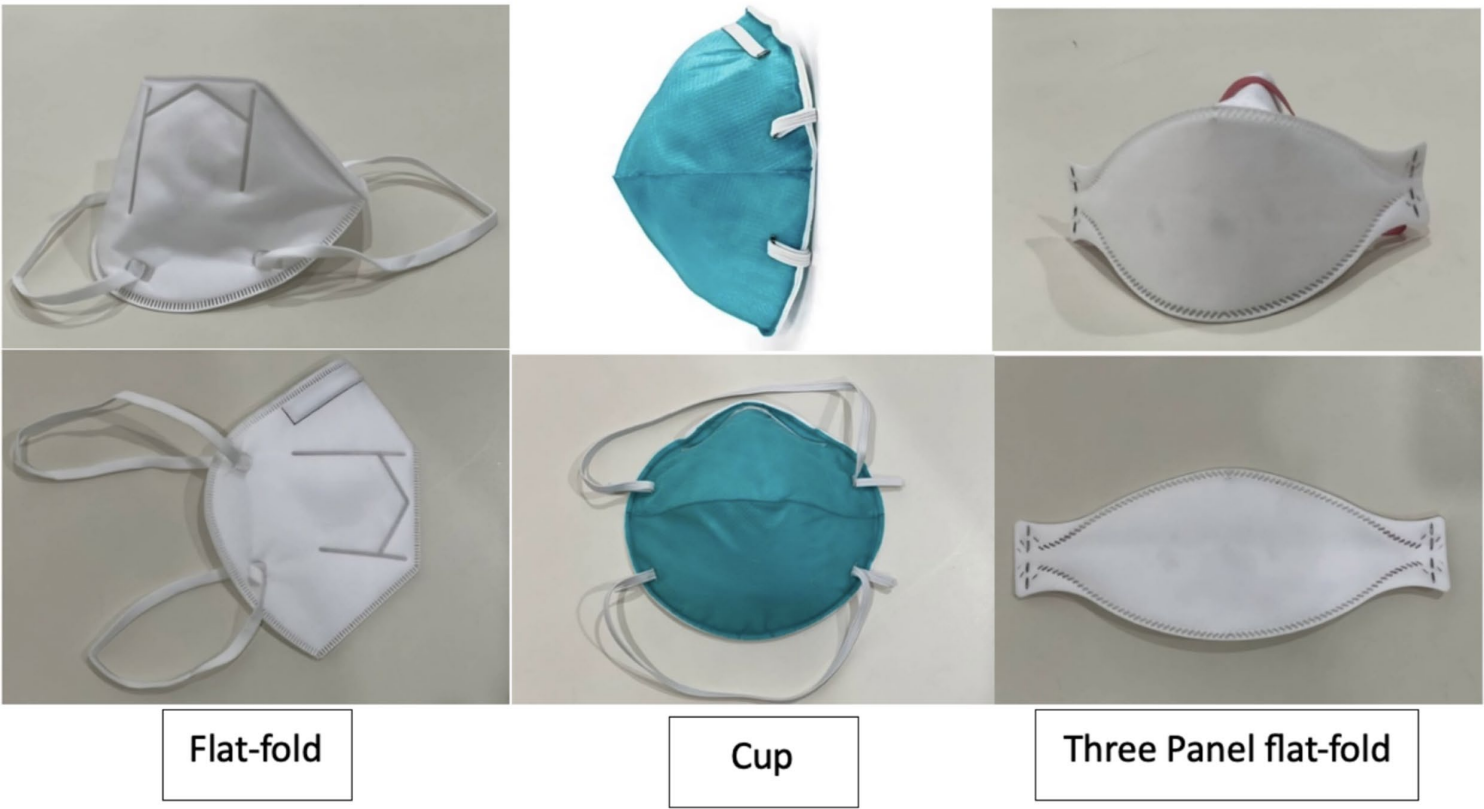
The filtering facepiece shapes.

The 22 facial anthropometric dimensions were selected based on the study by Lee et al. (25), which was extensively reviewed and selected from 109 dimensions related to half-face mask design. The 22 measured dimensions were as follows: head height, head breadth, head circumference, face length, lower face length, sellion-bottom lip length, bottom lip-menton length, nasal bridge-menton length, nasal bridge-chin length, chin-menton length, nose length, nose protrusion, face width, chin width, maximum nasal bridge breadth, nose width, lip width, bitragion-menton arc, bitragion-subnasal arc, bizygomatic menton arc, and menton-chin arc (Figure 1). The measurement was collected by spreading caliper, sliding caliper, and measuring tape.

### 2.5 Statistical analysis

**Figure 2:**
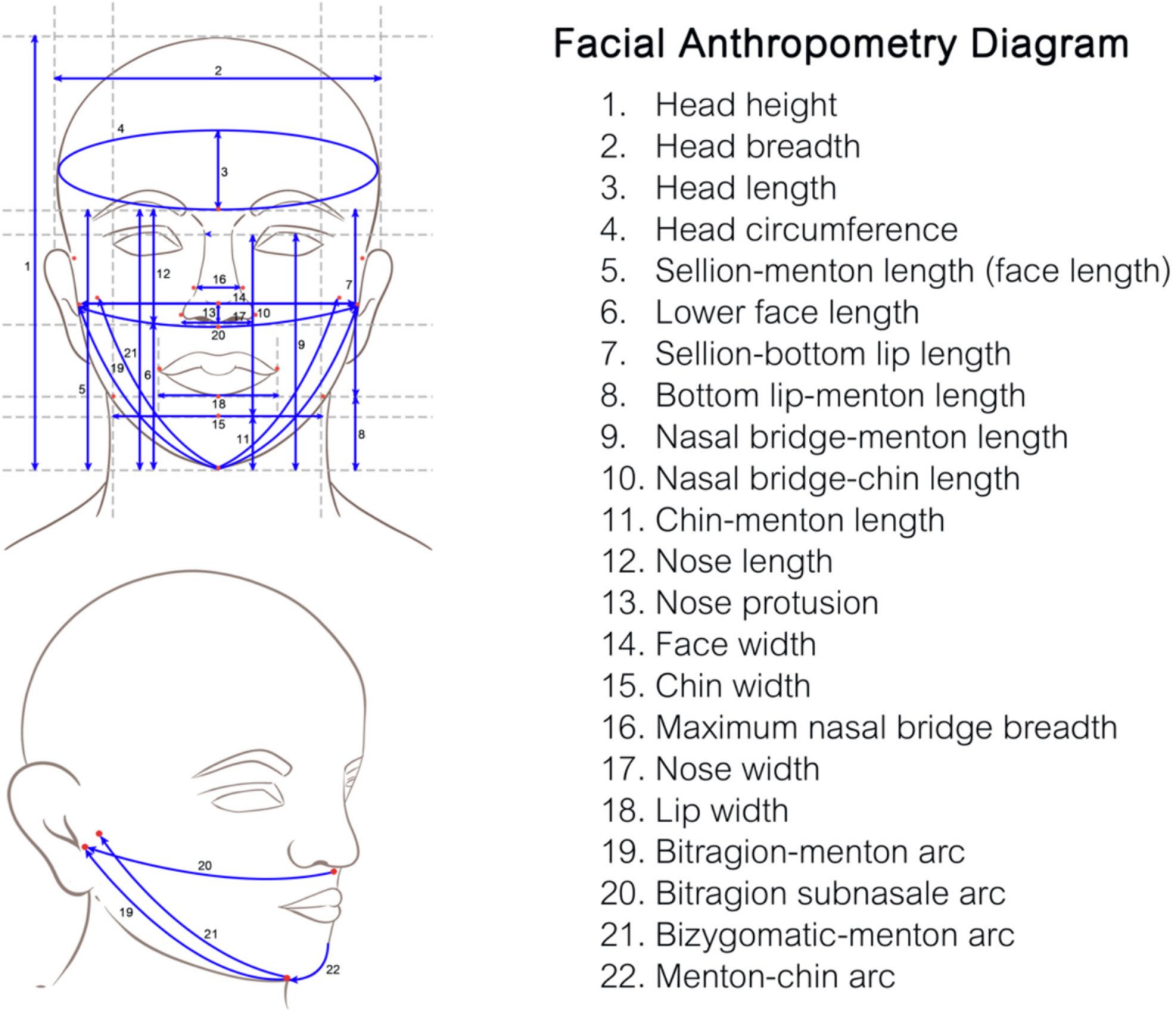
The facial anthropometric diagram showing the 22 measured dimensions, which were selected, adapted, and illustrated based on the study by Lee et al. (25).

Descriptive analysis was used to describe the fit factor and pass rate of each FFR. ANOVA or the Kruskal–Wallis test was used to analyze the difference in pass rates between each FFR. Fischer’s exact test and the *t*-test were used to evaluate the association of fit test passing and characteristic or anthropometric dimensions as appropriate. Multiple logistic regression was used to determine the degree of association between the fit test passing and anthropometric dimensions after adjusting the general characteristics, expressed as the adjusted odds ratio (Adj.OR) with 95% confidence intervals (CIs) and *p*-value (see Figure 2).

## 3 Results

### 3.1 The fit pass rates in different FFRs

**Figure 3:**
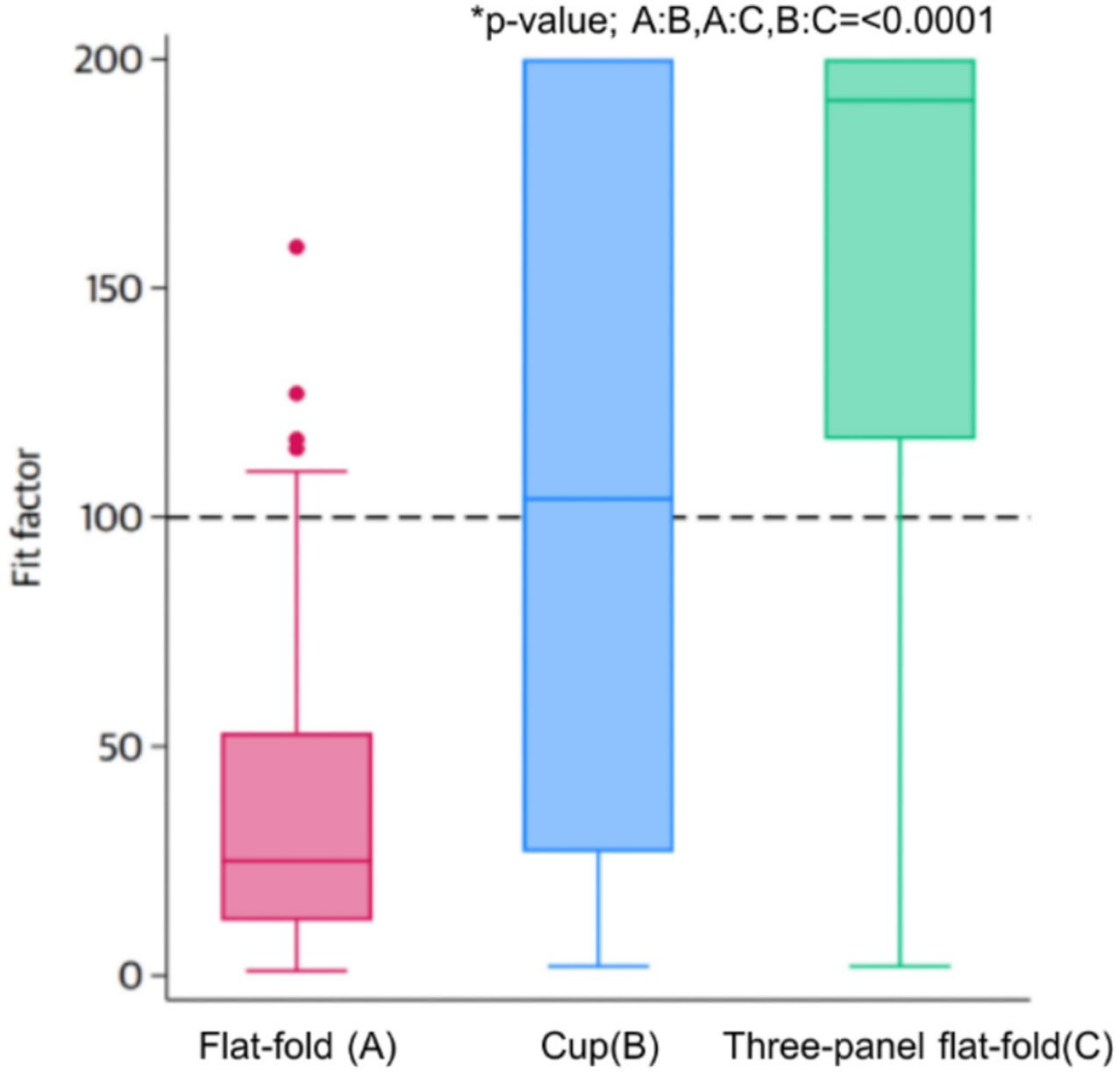
The overall fit factor in different FFRs.

The overall fit pass rate for all three FFRs was 86.9%. The pass rates for individual models were as follows: 5.4% for the flat-fold model [median Fit Factor [FF]: 25; interquartile range (IQR): 12–53], 51.1% for the cup model (median FF: 104; IQR: 27–200), and 82.5% for the three-panel flat-fold model (median FF: 191; IQR: 117–200). The difference in pass rates among the three FFRs was statistically significant (*p* < 0.001) (Figure 3).

Among the participants, the majority were female nurses. Analysis of the characteristics of those who passed the fit test revealed no significant factors associated with passing the flat-fold FFR. However, male participants, those with beards or mustaches, and taller individuals were more likely to pass the fit test for the cup-shaped FFR. Younger participants and those with shorter work experience showed a higher likelihood of passing the fit tests for both the cup-shaped and three-panel flat-fold FFRs (Table 1).

**Table 1:** Characteristics of participants.

Characteristic	Flat-fold			Cup			Three-panel flat-fold		
	Pass *N* = 12	Not pass *N* = 211	*p*-value	Pass *N* = 114	Not pass *N* = 109	*p-*value	Pass *N* = 184	Not pass *N* = 39	*p*-value
Gender									
Male	2 (16.7%)	32 (15.2)	1.000	24 (21.0%)	10 (9.2)	0.016*	28 (15.2%)	6 (15.4)	1.000
Female	10 (83.3%)	179 (84.8%)		90 (79.0%)	99 (90.8)		156 (84.8%)	33 (84.6%)	
Job									
Physician	2 (16.67%)	21 (10.0%)	0.883	15 (13.2%)	8 (7.3)	0.693	22 (12.0%)	1 (2.6%)	0.223
Nurse	6 (50.0%)	108 (51.2%)		57 (50.00)	57 (52.3%)		92 (50.0%)	22 (56.4%)	
Nurse aid	4 (33.3%)	69 (32.7%)		35 (30.7%)	38 (34.9%)		59 (32.1%)	14 (35.9%)	
Laboratory staff	0 (0.0%)	11 (5.2%)		6 (5.3%)	5 (4.6%)		10 (5.4%)	1 (2.6%)	
Others	0 (0.0%)	2 (1.0%)		1 (0.9%)	1 (0.9%)		1 (0.5%)	1 (2.6%)	
Facial surgery									
Yes	2 (16.7%)	20 (9.5%)	0.482	13 (11.4%)	9 (8.3%)	0.504	17 (9.2%)	5 (12.8%)	0.553
No	10 (83.3%)	191 (90.5%)		101 (88.6%)	100 (91.7%)		167 (90.1%)	34 (87.2%)	
Facial hair									
Yes	0 (0%)	12 (5.7%)	1.000	11 (9.6%)	1 (0.9%)	0.005*	10 (5.4%)	2 (5.1%)	1.000
No	12 (100.0%)	199 (94.3%)		103 (90.4%)	108 (99.1)		174 (94.6%)	37 (94.9%)	
Denture									
Yes	1 (8.3%)	9 (4.3%)	0.432	4 (3.5%)	6 (5.5%)	0.532	7 (3.8%)	3 (7.7%)	0.385
No	11 (91.7%)	202 (95.7%)		110 (96.5%)	103 (94.5%)		177 (96.2%)	36 (92.3%)	
Age (years)	36.1 (2.8)	37.9 (0.8)	0.586	35.7 (1.0)	39.9 (1.1)	0.005*	36.8 (0.8)	42.4 (1.7)	0.004*
Working experience (years)	13.1 (2.7)	15.2 (0.8)	0.5219	12.8 (1.0)	17.6 (1.1)	0.001*	14.3 (0.8)	19.1 (1.7)	0.016*
Height (cm)	160.9 (2.5)	158.92 (0.7)	0.511	160.9 (0.8)	157.1 (1.1)	0.006*	159.1 (0.8)	158.6 (1.2)	0.793
Weight (kg)	57.4 (3.5)	60.5 (0.9)	0.800	61.9 (1.33)	58.9 (1.1)	0.092	60.3 (0.9)	60.7 (2.6)	0.890
BMI (M/cm^2^)	22.0 (0.9)	24.6 (0.8)	0.480	23.8 (0.4)	25.1 (1.6)	0.412	24.5 (1.0)	24.0 (0.9)	0.830

* *p*-value <0.05.

**Figure 4:**
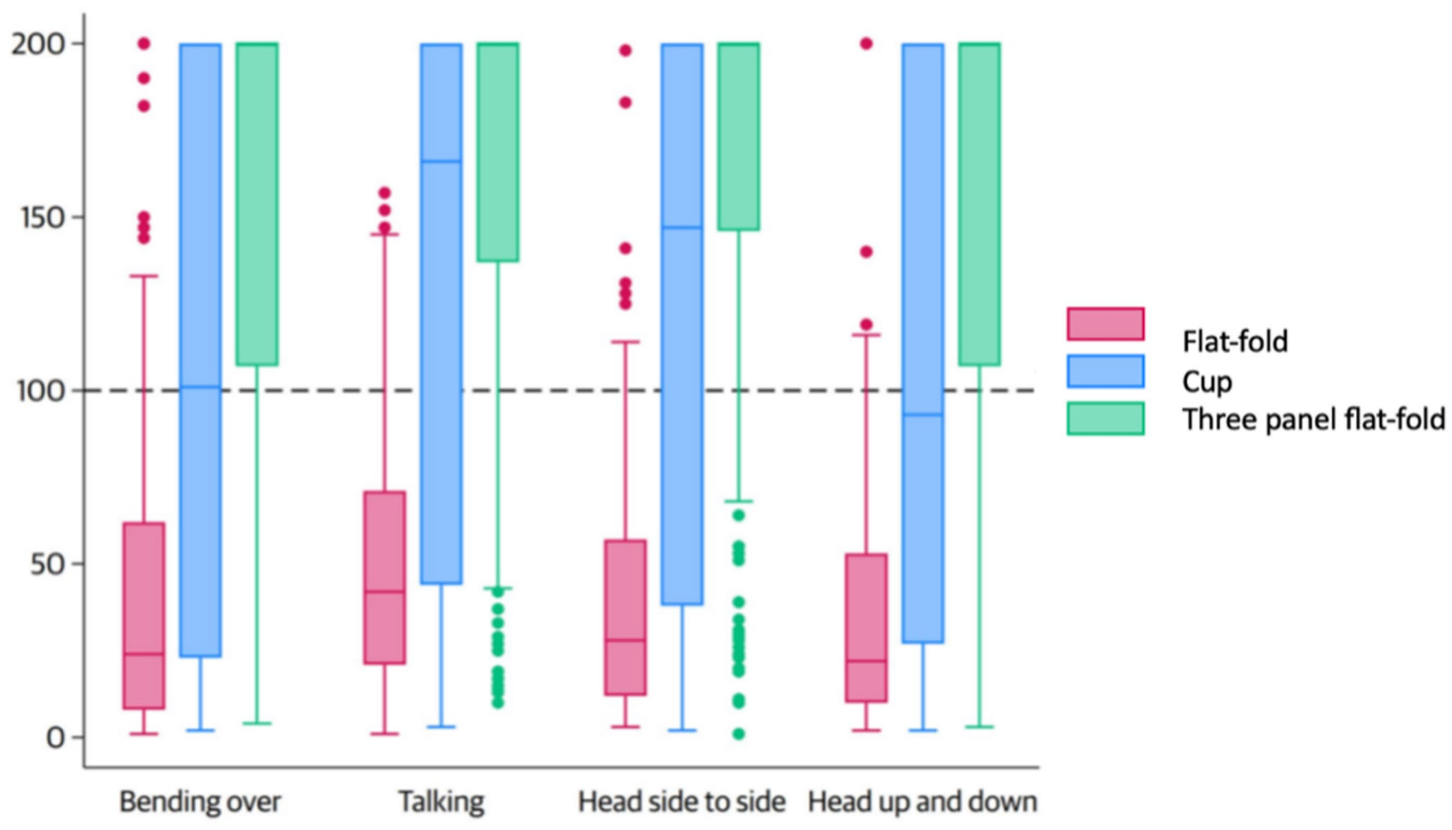
Fit factor in each position among different FFRs.

The FF values for the flat-fold, cup, and three-panel flat-fold models varied across the four fit test exercises. During the bending exercise, the FF values were 24 (IQR: 8–62), 101 (IQR: 23–200), and 200 (IQR: 107–200). In the talking exercise, the values were 42 (IQR: 21–71), 168 (IQR: 44–200), and 200 (IQR: 137–200). For the head side-to-side movement, the values were 28 (IQR: 12–57), 146 (IQR: 38–200), and 200 (IQR: 146–200). Finally, during the head up-and-down exercise, the FF values were 22 (IQR: 10–53), 93 (IQR: 27–200), and 200 (IQR: 97–200) (Figure 4).

### 3.2 Anthropometric influences on mask fit

The average measurements of 22 anthropometric dimensions among participants who passed and did not pass the fit tests for each mask type are summarized in Table 2. Significant dimensions associated with passing the fit test for the flat-fold FFR included head length, nose length, menton-chin arc, and sellion-bottom-lip length. For the cup-shaped FFR, maximum nasal bridge breadth emerged as a significant factor, while head length was associated with passing the fit test for the three-panel flat-fold FFR.

**Table 2:** Characteristics of anthropometric dimension.

Characteristic	Flat-fold			Cup			Three-panel flat-fold		
	Pass *N* = 12	Not pass *N* = 211	*p*-value	Pass *N* = 114	Not pass *N* = 109	*p*-value	Pass *N* = 184	Not pass *N* = 39	*p*-value
1. Head height	221.9 (12.8)	217.2 (13.9)	0.250	217.6 (13.8)	217.2 (14.0)	0.830	216.9 (14.0)	220.0 (13.2)	0.200
2. Head breadth	121.4 (7.4)	122.8 (7.3)	0.520	122.1 (7.8)	123.4 (6.8)	0.180	122.3 (7.3)	124.9 (7.2)	0.040*
3. Head length	181.4 (7.4)	176.3 (11.8)	0.140	176.7 (11.9)	176.5 (11.4)	0.890	175.9 (11.5)	179.8 (11.8)	0.055
4. Head circumference	543.2 (18.0)	544.9 (22.9)	0.800	547.4 (23.6)	542.2 (21.4)	0.080	544.6 (23.2)	545.7 (20.0)	0.780
5. Sellion-Menton length (face length)	116.0 (8.7)	111.0 (9.3)	0.069	111.4 (9.0)	111.1 (9.7)	0.800	111.1 (9.4)	111.9 (9.3)	0.640
6. Lower face length	60.6 (5.2)	60.5 (6.5)	0.930	60.9 (7.5)	60.0 (5.0)	0.260	60.8 (6.6)	59.1 (5.5)	0.150
7. Sellion-Bottom lip length	40.0 (2.5)	37.5 (5.0)	0.092	37.9 (4.5)	37.5 (5.3)	0.540	37.5 (4.9)	38.3 (5.1)	0.390
8. Bottom lip-Menton length	30.2 (6.5)	28.0 (4.4)	0.100	28.5 (4.5)	27.8 (4.5)	0.310	28.3 (4.6)	27.3 (4.0)	0.220
9. Nasal bridge-Menton length	101.3 (13.1)	94.1 (12.5)	0.053	95.3 (11.7)	93.6 (13.4)	0.330	94.6 (12.6)	93.7 (12.7)	0.690
10. Nasal bridge-Chin length	93.5 (10.9)	86.5 (11.7)	0.042	87.5 (10.8)	86.2 (12.6)	0.400	86.9 (11.1)	86.3 (14.5)	0.770
11. Chin-Menton length	19.9 (4.4)	18.8 (4.5)	0.380	18.9 (4.5)	18.7 (4.4)	0.710	18.8 (4.5)	18.9 (4.1)	0.920
12. Nose length	91.9 (8.7)	84.8 (11.2)	0.032*	85.7 (10.2)	84.7 (12.2)	0.510	84.9 (11.3)	86.4 (10.6)	0.450
13. Nose protrusion	14.6 (4.6)	13.8 (4.2)	0.500	13.6 (3.8)	14.2 (4.6)	0.240	13.8 (4.0)	14.6 (4.8)	0.280
14. Face width	104.9 (6.3)	107.5 (7.4)	0.240	107.1 (7.5)	107.7 (7.2)	0.570	107.1 (7.4)	108.6 (7.0)	0.250
15. Chin width	103.8 (7.8)	107.7 (9.5)	0.160	108.2 (9.1)	106.7 (9.8)	0.250	107.6 (9.5)	106.8 (9.2)	0.650
16. Maximum nasal bridge breadth	21.5 (3.0)	21.8 (3.4)	0.760	22.1 (3.4)	21.4 (3.4)	0.140	21.8 (3.3)	21.7 (3.9)	0.870
17. Nose width	37.1 (3.5)	38.1 (4.1)	0.440	38.0 (3.3)	38.0 (4.8)	0.960	38.0 (4.3)	38.3 (3.4)	0.680
18. Lip width	48.0 (3.6)	49.8 (5.3)	0.270	49.5 (4.6)	49.9 (5.9)	0.610	49.6 (5.3)	50.2 (5.3)	0.480
19. Bitragion-Menton arc	300.0 (18.2)	303.0 (17.4)	0.570	304.2 (18.2)	301.4 (16.4)	0.210	302.4 (17.4)	304.4 (17.2)	0.520
20. Bitragion subnasal arc	274 (10.2)	278.8 (14.4)	0.250	279.6 (15.0)	277.6 (13.6)	0.270	278.2 (14.2)	280.6 (14.4)	0.320
21. Bizygomatic-Menton arc	224.4 (22.4)	225.6 (16.8)	0.810	224.8 (16.6)	226.2 (17.6)	0.500	225.2 (17.0)	227.0 (17.6)	0.540
22. Menton-Chin arc	25.7 (5.7)	26.4 (6.5)	0.700	26.2 (6.5)	26.5 (6.4)	0.740	26.1 (6.5)	27.5 (6.0)	0.240

* *p*-value <0.05.

Significant associations were observed after adjustment for personal characteristics in the multiple logistic regression analysis. For the flat-fold FFR, head length (Adj. OR = 1.16, *p* = 0.037), sellion-bottom lip length (Adj. OR = 1.70, *p* = 0.023), bottom lip-menton length (Adj. OR = 1.45, *p* = 0.039), and nose length (Adj. OR = 1.28, *p* = 0.029) were significantly positively associated with passing the fit test. Conversely, the menton-chin arc (Adj. OR = 0.73, *p* = 0.019) was significantly negatively associated with passing the fit test. For the cup-shaped FFR, maximum nasal bridge breadth showed a significant association (Adj. OR = 1.11, *p* = 0.037). The three-panel flat-fold FFR demonstrated fewer negatively significant predictors, with head length showing a borderline association (Adj. OR = 0.95, *p* = 0.022) (Table 3).

**Table 3:** The association of anthropometric dimension with mask fit.

Characteristic	Flat-fold					Cup					Three-panel flat-fold				
	Adj. OR#	95%CI			*p*-value	Adj. OR#	95%CI			*p*-value	Adj. OR#	95%CI			*p*-value
1.Head height	0.98	0.91	–	1.07	0.765	0.97	0.95	–	1	0.089	0.96	0.93	–	1	0.075
2.Head breadth	1.02	0.86	–	1.23	0.771	0.97	0.91	–	1.03	0.289	0.92	0.85	–	1.01	0.082
3.Head length	1.16	1.01	–	1.36	0.037*	0.97	0.95	–	1.01	0.151	0.95	0.91	–	0.99	0.022*
4.Head circumference	0.97	0.91	–	1.03	0.369	1.00	0.99	–	1.02	0.667	1.00	0.98	–	1.03	0.679
5.Sellion-Menton length (face length)	0.89	0.76	–	1.05	0.190	0.96	0.9	–	1.03	0.232	1.00	0.92	–	1.07	0.838
6.Lower face length	0.84	0.67	–	1.06	0.147	1.00	0.94	–	1.06	0.978	1.03	0.95	–	1.12	0.463
7.Sellion-Bottom lip length	1.70	1.08	–	2.7	0.023*	0.98	0.92	–	1.06	0.642	0.96	0.88	–	1.07	0.51
8.Bottom lip-Menton length	1.45	1.02	–	2.07	0.039*	1.02	0.92	–	1.12	0.755	1.05	0.92	–	1.2	0.475
9.Nasal bridge-Menton length	0.98	0.84	–	1.16	0.868	1.00	0.95	–	1.06	0.813	1.00	0.95	–	1.07	0.776
10.Nasal bridge-Chin length	1.18	0.92	–	1.52	0.190	1.00	0.95	–	1.06	1.000	1.00	0.9	–	1.07	0.827
11.Chin-Menton length	0.86	0.67	–	1.13	0.290	1.00	0.92	–	1.1	0.872	1.00	0.9	–	1.12	0.948
12.Nose length	1.28	1.03	–	1.61	0.029*	1.03	0.99	–	1.09	0.128	0.98	0.92	–	1.04	0.535
13.Nose protrusion	0.80	0.49	–	1.3	0.371	0.85	0.71	–	1.01	0.068	0.94	0.76	–	1.17	0.598
14.Face width	0.96	0.78	–	1.18	0.665	1.00	0.94	–	1.07	0.975	1.00	0.92	–	1.03	0.946
15.Chin width	0.89	0.76	–	1.05	0.190	1.00	0.96	–	1.05	0.957	1.03	0.89	–	1.1	0.200
16.Maximum nasal bridge breadth	0.92	0.73	–	1.16	0.487	1.11	1.01	–	1.24	0.037*	1.02	0.94	–	1.16	0.714
17.Nose width	1.15	0.89	–	1.5	0.281	0.97	0.88	–	1.08	0.619	1.05	0.9	–	1.17	0.335
18.Lip width	0.79	0.62	–	1.02	0.074	0.98	0.92	–	1.07	0.793	0.99	0.95	–	1.09	0.828
19.Bitragion-Menton arc	1.02	0.94	–	1.10	0.628	1.00	0.97	–	1.03	0.894	1.00	0.96	–	1.03	0.901
20.Bitragion subnasal arc	0.89	0.77	–	1.02	0.094	1.00	0.98	–	1.05	0.495	0.99	0.94	–	1.04	0.69
21.Bizygomatic-Menton arc	0.94	0.87	–	1.01	0.129	1.01	0.96	–	1.00	0.108	1.01	0.98	–	1.1	0.447
22.Menton–Chin arc	0.73	0.57	–	0.95	0.019*	1.03	0.97	–	1.11	0.285	1.00	0.91	–	1.09	0.936

^#^Adjusted Odd ratio for height, weight, facial surgery, working experience, gender, age, beard and mustache, and, denture. *p-value <0.05.

## 4 Discussion

### 4.1 The fit pass rates

The highest fit test pass rate of the current study was observed for the three-panel flat-fold model, while the lowest was for the flat-fold model. However, the overall fit test pass rate, defined as passing at least one out of three respirators tested, in the current study was 86.9%. This is relatively high compared to a systematic review of respirator use during the COVID-19 pandemic, which reported that 67.86% of studies observed fit pass rates exceeding 50%. Among those studies, the highest pass rate recorded was 98.2%, while the lowest was 1.1%. These findings highlight the considerable variability in fit test outcomes for FFRs and emphasize the critical importance of appropriate selection and fitting to achieve effective respiratory protection (6). The fit test pass rate is influenced by various factors, including respirator type, user demographics, and working conditions (12). Offering multiple respirator options may also increase the likelihood of finding a suitable fit for each participant. Our study demonstrates differing fit pass rates across three types of FFRs, each yielding distinct pass rates in various directions. This variation underscores the importance of strategic planning in providing appropriate FFRs for the Thai or Southeast Asian population. By analyzing the fit effectiveness of multiple respirator types, we can better tailor facemask provisions to enhance respiratory protection among diverse user groups in the Thai or Southeast Asian population. The current study has conducted three different shapes of the respirator, which is commonly used in Thailand. The variability in fit pass rates across masks warrants further investigation in diverse settings, which is important for the fundamental designing of the best-fit shape FFRs in the future.

Our study reported a pass rate of 5.4% for flat-fold masks, with a median FF of 25 ± 30.8, which is considerably lower than the results from other studies. For instance, studies from other regions in Australia report pass rates ranging from 18 to 57% for different flat-fold mask models (8, 26). The study in Asia also reported a higher pass rate. Zhang et al. (16) reported a significantly higher pass rate of 51.8% for two flat-fold FFR models tested in China, with FF values of 92.2 and 121. Similarly, the study from Hong Kong showed a high pass rate for flat-fold nanofiber N95 at 78.8% (27). The most similar report to our study was a study in Iran, which also demonstrated a low pass rate of 9% across 14 flat-fold mask models, with rates ranging from 0 to 27% and an average FF of 30 (19).

In comparison, the cup-shaped FFR in our study demonstrated a higher pass rate of 51.1%, with a median FF of 104 ± 77.3, outperforming flat-fold masks. Our result was similar to that of other studies from Asia. A study conducted in Malaysia reported a slightly higher pass rate of 57%, with a geometric mean (GM) FF of 79.64 ± 2.65. Similarly, Zhang et al. (16) reported a comparable pass rate of 57.1% (FF 121.5) among Chinese participants. The current highest pass rate among the Asian studies was reported in Hong Kong at 60.8% (28). However, the study by Fakherpour et al. (19) in Iran observed a much lower pass rate of 16.2%, with an average FF of 48. Studies from other regions have reported higher pass rates; for instance, a study by Ng et al. from Australia reported a higher pass rate of 65% (16, 18, 26).

The three-panel flat-fold respirator achieved the highest pass rate in our study, at 82.5% (median FF 191 ± 58.7), which is in concordance with the other studies from Asia that showed 74.1 and 65.0% pass rates reported in 135 Malaysian samples (including Malays, Chinese, and Indians) (GM FF 122.24 ± 2.50) and in 638 Hong Kong Chinese samples of healthcare students, respectively (18, 28). A Canadian study on a multi-ethnic population correspondingly reported a high pass rate of 75% for three-panel flat-fold masks (29). Notably, some studies from other regions reported near-perfect pass rates, such as those by Ng et al. (26) at 96.4% and Williams et al. (30, 31) at 99.2%, with all tested mask types achieving FF values above 166. Variations in fit pass rates across studies may be attributed to differences in sample demographics, testing protocols, or mask models used.

The findings of this study reveal significant differences in fit test pass rates across respirator shapes, with the three-panel flat-fold design outperforming cup-shaped and flat-fold masks. This trend aligns with previous studies, which often report the highest pass rates for three-panel flat-fold designs (32). The superior performance of three-panel designs may be attributed to their greater adaptability to diverse facial shapes, whereas the semi-rigid structure of cup-shaped FFRs and the limited vertical length of flat-fold FFRs may hinder their adaptability (32). However, mask design should not only prioritize fit but also consider other essential factors, such as wearer comfort, durability, and overall usability. Balancing these properties is critical to the development of effective and user-friendly respirators.

### 4.2 Anthropometric influences on mask fit

This study is the first in Thailand to evaluate facial anthropometric dimensions, an important factor influencing mask fit, as highlighted in several previous studies (12, 16, 17, 29, 33–37). The National Institute of Occupational Health (NIOSH) developed a Respiratory Fit Test Panel (RFTP) to ensure that respirators provide effective protection across diverse facial dimensions. The NIOSH RFTP employs bivariate analysis of two primary facial measurements—face length (menton-sellion length) and face width (bizygomatic breadth)—and applies Principal Component Analysis (PCA) to 10 facial dimensions significantly associated with respirator fit (11).

The current study, however, identified that the fit of different mask shapes may be associated with distinct anthropometric dimensions. Specifically, four dimensions—head length, chin-menton length, sellion-to-bottom-lip distance, and nose length—were associated with flat-fold FFR fit passing. In contrast, the cup-shaped FFR and three-panel flat-fold FFR were each associated with a single dimension, maximum nasal bridge breadth, and head length, respectively. In contrast, the study by Khairul Hasni et al. (18), conducted in Malaysia, identified significant positive associations between fit factors of various FFRs and facial dimensions such as nose protrusion, nasal root height, and the subnasale-sellion distance. Conversely, negative associations were observed with dimensions such as menton-sellion distance, bigonial breadth, and nose breadth (18). Similarly, the study by Zhang et al. (16) reported results that differed from the current study but partially aligned with the NIOSH bivariate model. Their findings indicated that fit rates were positively influenced by facial length, whereas a negative impact was observed for the bitragion-submandibular arc (16). The suitability of the NIOSH RFTP for non-Western populations remains inconclusive, even in studies conducted within the same region. For instance, Chen et al. (38) found that 95% of workers in a Chinese survey fell within the NIOSH bivariate and PCA parameters, although the distribution of participants across the panel was uneven. Similarly, Yang et al. (39) reported that the facial dimensions of Chinese individuals tend to be shorter and wider than the American dimensions defined by the NIOSH RFTP. Moreover, Seo et al.’s (17) study in the Republic of Korea revealed that the fit test results did not vary when using the NIOSH facial categories, suggesting that the NIOSH bivariate panel does not adequately represent the facial sizes of Korean HCP.

Locally, there is limited evidence on head and facial anthropometric data to inform the development of face mask designs suitable for the Thai population. Furthermore, no relevant data are available from neighboring countries, apart from a single study conducted on a multi-ethnic population in Malaysia (40). This study provides preliminary anthropometric data, highlighting the need to evaluate the mask design for the Thai population. While further research is required, the findings indicate that the three-panel flat-fold design offers better-fit potential and may inform regionally optimized respirator design. These results also underscore the importance of developing a localized RFTP tailored to the facial characteristics of the Thai population. Such efforts could ensure that mask designs provide an adequate fit for local users and may also support the creation of a regional RFTP to address the needs of Southeast Asian populations with similar facial dimensions (41). Future research should focus on validating localized RFTPs and assessing their effectiveness in improving mask fit for Southeast Asian populations.

### 4.3 Head movements and leakage

The fit testing also provided data on various positions representing typical activities during mask usage, including breathing, bending, head movements (up-down and side-to-side), and talking (20). The results revealed a trend of lower fit factor during bending and head-up or head-down movements. While specific studies detailing the exact reasons for lower fit factors during the bending-over exercise are limited, the consensus is that movements causing significant changes in head and neck positions, such as bending over, can affect respirator fit (42). The protective efficiency of masks diminishes with head movements, such as bending, speaking, lateral and vertical motions, and facial expressions such as grimacing. These actions disrupt the seal by altering the interface between the mask and the wearer’s face, leading to additional leakage points. The degree of efficiency loss is strongly influenced by the quality of the initial fit and the compatibility between the mask design and the wearer’s facial anatomy (43–45). Identifying leakage points is essential for improving the fit of filtering facepiece respirators (FFRs). Prior research utilizing head-form simulations for cup-shaped FFRs has identified the nose clip as the most common leakage site (46). Consistent with these findings, our study observed that individuals with a wider nasal bridge demonstrated significantly higher pass rates with cup-shaped FFRs. This could be due to a reduced need for molding around the nose area, resulting in a better seal than individuals with narrower nasal bridges. Therefore, mask design processes should prioritize adjustments around the nose clip to ensure it can accommodate variations in nasal dimensions across the target population.

## 5 Strengths and limitations

This study is the first to compare the fit pass rates of different FFR designs among Thai HCP and provides preliminary anthropometric data specific to this population. These findings offer a foundation for developing improved respirator designs and a localized respiratory fit test panel tailored to the Thai population. The study addresses a critical issue in Southeast Asia, where respirators are often imported and not optimized for local facial features, making the results regionally significant.

However, the study has several limitations. It was conducted in a single university hospital, which may limit the generalizability of the findings to HCP in other healthcare settings. The sample predominantly consisted of Thai HCP, potentially limiting the applicability of the findings to multi-ethnic populations or regions with greater facial diversity. Additionally, only three NIOSH-certified N95 respirator models were tested, which may not represent the full range of respirators available in the market. The study focused exclusively on fit without assessing key factors such as comfort, breathability, and usability—which play a critical role in ensuring user compliance (47). Furthermore, the cross-sectional design captures a single point in time without accounting for changes in fit over prolonged use.

Future studies should adopt a multi-center design to include HCP from various institutions across Thailand, enhancing the generalizability of the findings. Expanding to diverse healthcare settings, such as rural hospitals and private clinics, would provide a broader representation of workplace conditions and demographics.

Longitudinal studies are needed to assess changes in fit over time and the impact of repeated use. Research should also explore fit testing among vulnerable populations, such as children and older adult individuals, and evaluate usability and comfort in real-world conditions.

Additionally, developing a regional respiratory fit test panel specific to Southeast Asia is crucial to guide respirator design and ensure optimal protection for diverse user groups.

## 6 Conclusion

This study provides valuable insights into the fit test performance of three commonly used disposable respirator designs in Thailand: flat-fold, cup-shaped, and three-panel flat-fold. Among them, the three-panel flat-fold design showed the highest fit test pass rate, followed by the cup-shaped and flat-fold designs, likely due to its greater adaptability to various facial shapes. These findings highlight the potential benefit of considering local anthropometric variations in respirator design and the need for further investigation into the applicability of existing global standards, such as those developed by NIOSH, to Thai and Southeast Asian populations.

The study also identified key factors influencing fit, including head movements and specific facial dimensions, particularly around the nasal bridge, which is a common site for leakage. These observations suggest that improving design features, such as the adjustability of the nose clip, could enhance fit and protective efficiency. While the results suggest that providing multiple respirator options may increase the likelihood of achieving a proper fit, further research is needed to confirm these findings and explore the development of localized Respiratory Fit Test Panels (RFTPs) tailored to the facial dimensions of the Thai population. This study contributes preliminary data that may inform future efforts to optimize respirator fit in Thailand and neighboring regions.

## Data Availability

The raw data supporting the conclusions of this article will be made available by the authors, without undue reservation.
